# Gut and respiratory tract microbiota in children younger than 12 months hospitalized for bronchiolitis compared with healthy children: can we predict the severity and medium-term respiratory outcome?

**DOI:** 10.1128/spectrum.02556-23

**Published:** 2024-05-24

**Authors:** Raul Cabrera-Rubio, Cristina Calvo, Sonia Alcolea, María Bergia, Jorge Atucha, Francisco Pozo, Inmaculada Casas, María Arroyas, Maria Carmen Collado, Maria Luz García-García

**Affiliations:** 1 Department of Biotechnology, Institute of Agrochemistry and Food Technology–National Research Council (IATA-CSIC), Paterna, Valencia, Spain; 2 Paediatric Infectious Diseases Department, La Paz University Hospital, Madrid, Spain; 3 La Paz University Hospital Institute for Health Research (IdiPAZ), Madrid, Spain; 4 Translational Research Network in Paediatric Infectious Diseases (RITIP), Madrid, Spain; 5 CIBER of Infectious Diseases (CIBERINFEC ISCIII), Madrid, Spain; 6 Respiratory Viruses and Influenza Unit at the National Centre for Microbiology ISCIII (CIBERESP ISCIII), Madrid, Spain; 7 Paediatric Department, Severo Ochoa University Hospital, Leganés, Madrid, Spain; 8 Instituto de Investigación Sanitaria Puerta de Hierro Segovia de Arana (IDIPHISA), Madrid, Spain; University of Georgia, Athens, Georgia, USA

**Keywords:** infants, virus, respiratory syncytial virus, nasopharyngeal microbiota, gut microbiota

## Abstract

Growing evidence indicates that gut and respiratory microbiota have a potential key effect on bronchiolitis, mainly caused by respiratory syncytial virus (RSV). This was a prospective study of 96 infants comparing infants with bronchiolitis (*n* = 57, both RSV and non-RSV associated) to a control group (*n* = 39). Gut (feces) and respiratory [nasopharyngeal aspirate (NPA)] microbial profiles were analyzed by 16S rRNA amplicon sequencing, and respiratory viruses were identified by PCR. Clinical data of the acute episode and follow-up during the first year after infection were recorded. Pairwise comparisons showed significant differences in the gut (*R*^2^ = 0.0639, *P* = 0.006) and NPA (*R*^2^ = 0.0803, *P* = 0.006) microbiota between cases and controls. A significantly lower gut microbial richness and an increase in the NPA microbial diversity (mainly due to an increase in *Haemophilus*, *Streptococcus,* and *Neisseria*) were observed in the infants with bronchiolitis, in those with the most severe symptoms, and in those who subsequently developed recurrent wheezing episodes after discharge. In NPA, the higher microbial richness differed significantly between the control group and the non-RSV bronchiolitis group (*P* = 0.01) and between the control group and the RSV bronchiolitis group (*P* = 0.001). In the gut, the richness differed significantly between the control group and the non-RSV group (*P* = 0.01) and between the control group and the RSV bronchiolitis group (*P* = 0.001), with higher diversity in the RSV group. A distinct respiratory and intestinal microbial pattern was observed in infants with bronchiolitis compared with controls. The presence of RSV was a main factor for dysbiosis. Lower gut microbial richness and increased respiratory microbial diversity were associated with respiratory morbidity during follow-up.

**IMPORTANCE:**

Both the intestinal and respiratory microbiota of children with bronchiolitis, especially those with respiratory syncytial virus infection, are altered and differ from that of healthy children. The microbiota pattern in the acute episode could identify those children who will later have other respiratory episodes in the first year of life. Preventive measures could be adopted for this group of infants.

## INTRODUCTION

Respiratory infections occur most frequently in children and are the leading cause of consultation and hospitalization for children younger than 5 years (1). The infections are mostly caused by respiratory viruses, with respiratory syncytial virus (RSV) being the most frequent agent and responsible for a large burden of disease at this age (2, 3). Annual RSV epidemics represent a public health problem in children younger than 1 year, causing disease in 1%–2% of infants, mainly generating symptoms of bronchiolitis and requiring hospitalization in approximately 3%–10% of cases (3). Other respiratory viruses, such as human rhinovirus (HRV), human metapneumovirus, human adenovirus, human bocavirus, and human coronavirus (HCoV), alone or in coinfection, are also causal agents of respiratory disease in the first years of life (4–7). Severe respiratory infections requiring hospitalization, especially those associated with RSV and HRV coinfection, are frequently associated with mid- to long-term respiratory morbidity, with subsequent development of recurrent wheezing and even asthma in later life (8, 9). However, the reasons why certain children have more serious infections or have a special susceptibility to developing wheezing are not completely known. This different progression is possibly motivated by a complex interaction between the respiratory mucosa, innate and adaptive immunity, viral evasion strategies, and certain environmental factors. The role of the microbiota, both intestinal and respiratory, in the risk of severe lower respiratory tract infection or in subsequent respiratory morbidity, is under research. Several studies have compared the fecal microbiota in hospitalized children with bronchiolitis and in healthy children. In a case-control study, Hasegawa et al. (10) identified four distinct fecal microbiota profiles in infants, with the *Bacteroides*-dominant profile being associated with a higher likelihood of bronchiolitis. In the same population, the authors also identified four distinct nasal airway microbiota profiles. *Moraxella*-dominant and *Corynebacterium/Dolosigranulum*-dominant profiles were associated with a low likelihood of bronchiolitis, whereas a *Staphylococcus*-dominant profile was associated with a high likelihood of bronchiolitis (11). The authors concluded that interactions between RSV and nasopharyngeal microbiota might modulate the host immune response, potentially affecting clinical disease severity, a conclusion reached by other studies (12). Our main objective was to simultaneously study the intestinal and respiratory tract microbiota in infants younger than 12 months hospitalized for bronchiolitis in whom a virological diagnosis of the infection was established and to compare it with that of infants without identified viruses and with that of a control group of healthy infants. Our secondary goal was to evaluate the microbiota’s potential role in the development of recurrent wheezing in the year after discharge for bronchiolitis.

## MATERIALS AND METHODS

### Study population and design

This was a multicenter prospective study conducted in two hospitals in Madrid (Severo Ochoa University Hospital and La Paz University Hospital) between October 2018 and March 2020. Clinical data were collected in an anonymized database created for this purpose.

During the study period, we included all infants younger than 1 year (3.8 ± 3.0 months) admitted for bronchiolitis and all healthy infants younger than 1 year (6.1 ± 3.5 months) who attended pediatric outpatient visits (without viral infection). Infants who received treatment with antibiotics or glucocorticoids in the last 15 days were excluded from the study. In the healthy group, infants who had fever, respiratory, or gastrointestinal infection in the last 15 days were excluded.

During the hospital stay and as part of the study, a physician completed the study questionnaire with the following variables: age, sex, history of prematurity and underlying chronic diseases, need for oxygen therapy (provided to achieve transcutaneous oxygen saturation ≥ 94%), axillary temperature > 38°C, presence of infiltrates/atelectasis in radiographs, antibiotic therapy, length of hospital stay, and need for admission to an intensive care unit.

Both groups of infants (cases and controls) were followed up for 1 year after discharge, only for clinical monitoring. The parents were contacted monthly by phone, and an interview based on a structured questionnaire was performed to obtain information on wheezing episodes, related hospital admissions, chronic asthma treatment, physician-diagnosed atopic dermatitis, allergic rhinitis, food allergy, day care attendance, siblings, parental smoking habits, allergy, eczema, and asthma in first-degree family members diagnosed by a medical doctor.

Bronchiolitis was defined as the first episode of bronchospasm associated with upper respiratory tract infection in infants younger than 1 year or the presence of hypoxia (oxygen saturation < 94%, determined by pulse oximetry) and hyperinflation on chest X-rays in the absence of bronchospasm (McConnochie criteria) (13). Severe bronchiolitis was considered when oxygen therapy was required for more than 5 days or intensive care was required. Recurrent wheezing was defined as the presence of wheezing diagnosed by a doctor during the first 2 years of life.

### Biological sample collection: respiratory and fecal samples

Between 7 a.m. and 8 a.m. on the morning after the first day of admission, two respiratory samples were collected from the hospitalized patients by nasopharyngeal aspirate (NPA). One sample was kept refrigerated at 4°C until transportation to the National Centre for Microbiology (ISCIII), Spain, for the determination of specific virus in nasopharyngeal aspirates, and the other, to be used in the microbiota study, was stored immediately at −80°C until use. The respiratory samples of the control group were obtained during a clinical visit to the hospital, and samples followed the same procedure detailed above.

Fecal samples were collected through a standardized protocol at hospital admission (in cases) or at home the day before the clinic visit (in controls). Fecal samples were placed in sterile fecal collection containers and immediately stored at −80°C until analysis.

### Determination of specific virus in nasopharyngeal aspirates

The NPAs were sent for virological study to the Respiratory Viruses and Influenza Unit at the National Centre for Microbiology (ISCIII). Upon reception, three aliquots were prepared and stored at −80°C. RNA and DNA from 200 µL aliquots of NPA were extracted with the QIAamp Mini Elute Virus Spin Kit in an automated extractor (QIAcube, Qiagen, Valencia, CA, USA).

Detection of respiratory viruses was performed by four independent multiplex reverse transcription-polymerase chain reaction (RT-PCR) assays. The first assay detected influenza A, B, and C viruses, the second was used to detect parainfluenza viruses 1–4, HRVs, and enteroviruses, and the third assay detected the presence of respiratory syncytial virus types A and B, human metapneumovirus, human bocavirus, and human adenovirus. These three assays were real-time multiplex RT-PCRs and used the SuperScript III Platinum One-Step Quantitative RT-PCR System (Invitrogen). A fourth multiplex RT-PCR was employed to investigate HCoV, using generic primers that could detect both alpha and beta coronaviruses. Typing of HCoV was performed using a reverse specific primer for detecting HCoV 229E, HCoV NL63, HCoV OC43, and HCoV HKU1. Primers and TaqMan probes for the three independent multiplex real-time RT-PCRs were based on previously published designs by our group; the HCoV primers are available on request (14).

### Microbiota profiling

#### DNA extraction and 16S rRNA amplicon sequencing

Due to the potential of low microbial DNA in the infants’ NPA, specific quantitative PCR for total bacterial load was performed as previously described (15), before 16S amplicon sequencing. In brief, PCRs were performed in a LightCycler 480 Real-Time PCR System (Roche) using SYBR Green. The NPA samples with <10^4^ gene copies were excluded from the study due to low microbial biomass.

Total DNA was extracted from paired NPAs (500 µL) and fecal material (approximately 100 mg), employing an automated assisted method based on magnetic beads (Maxwell RSC Instrument coupled with Maxwell RSC Pure Food GMO and authentication kit; Promega, Spain), following the manufacturer’s instructions with previous treatments to improve the DNA quality and efficiency. In brief, samples were treated with lysozyme (20 mg/mL) and mutanolysin (5 U/mL) for 60 min at 37°C with a preliminary step of cell disruption with 3-μm-diameter glass beads for 1 min at 6 m/s by a FastPrep 24-5G Homogenizer bead beater (MP Biomedicals). The DNA obtained was purified with a DNA Purification Kit (Macherey-Nagel, Duren, Germany) according to the manufacturer’s instructions, and DNA concentration was measured with a Qubit 2.0 Fluorometer (Life Technology, Carlsbad, CA, USA) for further analysis.

Microbial profiles were determined by the V3–V4 variable region of the 16S rRNA gene sequencing, following PE250 Illumina strategy sequencing protocols. Amplicons were obtained with PCR amplification, using barcoded conventional primers (341F 5′-CCTACGGGNGGCWGCAG-3′ and 806R 5′GGACTACNNGGGTATCTAAT-3′) with a 466 bp fragment length. The libraries were generated with the NEBNext Ultra DNA Library Prep Kit from Illumina, and amplicons were checked with a Bioanalyzer DNA 1000 chip (Agilent Technologies, Santa Clara, CA, USA). The amplicons were sequenced on an Illumina paired-end platform to generate 250-bp paired-end raw reads on a NovaSeq-PE250 Illumina platform (Novogene Bioinformatics Technology Co., Ltd) according to the manufacturer’s instructions. Controls (negative and positive controls) during DNA extraction and PCR amplification were also included and sequenced.

#### Bioinformatics and statistical analyses

A bacterial diversity analysis was performed using raw reads, which were quality controlled and filtered (*Q* > 20 and length > 50 bp) using fastqc (v0.11.8) and trimGalore (v0.6.4_dev; https://github.com/FelixKrueger/TrimGalore). In addition, trimGalore was employed for adapter removal. The reads resulting from the previous procedure were processed with Quantitative Insights Into Microbial Ecology 2 (QIIME version 2018.11), and a standard 16S workflow was used for analysis (16). Sequences were joined with VSEARCH (17). Next, the reads were denoised with Deblur (18) and run with default parameters, with the exception of the minimum reads’ parameter set to 0 to account for metadata categories with smaller sample sizes and trim length set to 235 bases. Negative controls were also used to filter out contaminating amplicon sequence variants (ASVs) if present in each sample. Amplicon sequence variants were annotated with a Naive-Bayes classifier based on the scikit-learn system and the RDP database (19). The ASVs were aligned with MAFFT (20) to make a phylogenetic tree with FASTTREE (21), which was then midpoint rooted. The *P* values were adjusted using the false discovery rate method (based on the Benjamini–Hochberg procedure). *P* values < 0.05 were considered statistically significant.

The calculation of alpha-diversity indices, Chao1 and Shannon indices, for each sample was obtained by using the phyloseq R package (22), and differences by group were assessed by analysis of variance tests (non-significant associations *P* > 0.05 were not included in the figures). The PERMANOVA test (adonis2; terms added sequentially) from the vegan R package (23) evaluated overall differences in microbiota structure using a Bray–Curtis matrix of normalization data (log ratio) from abundance data of ASVs and vegan distance analysis. Rarefied was not performed, but a filter of 0.01% relative abundance in a minimum of 25% of the samples was established to perform the following analyses. In addition, a linear discriminant analysis effect size (LEfSe) was performed to discover specific bacterial biomarkers associated with health and disease states. A random forest classification model was built to identify the most discriminative ASVs between participants not exposed and exposed to antibiotics using the randomForest R package (package “randomForest*”*) with default parameters and including all ASVs assigned to the genus as explanatory variables as well as confounding variables as covariates. For a classification tree, variable importance was measured by the mean decrease in the GINI coefficient (measure of node purity) due to that variable, without being able to detect the most important association factors and genus level with either group (cases vs controls).

To verify these findings, a fuzzy set ordination was performed, which can be used as an alternative to test whether perturbation in any of the factors causes significant changes in the observed community structure. Ordination is a method for finding associations between site factors (without confounder factors) and species distributions. An alternative method for evaluating the environmental variables associated with the composition of the microbiota is a multivariate analysis used in ecology to relate the composition of samples with possible explanatory variables, such as FSO (Table S2). Another approximation is the cal_diff function, which is used to test the significance of variables across groups (Table S3).

## RESULTS

### Study population and design

**Fig 1:**
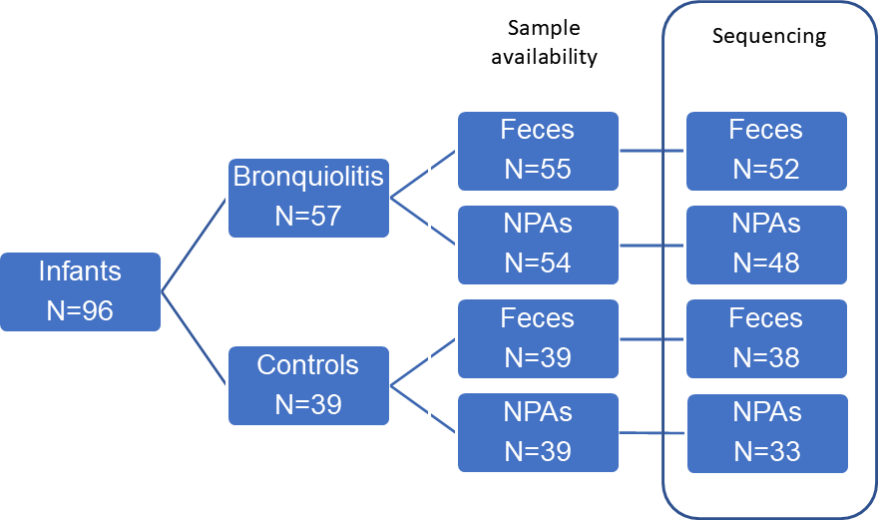
Patients’ and samples’ flow chart.

The study included 96 infants (57 hospitalized for bronchiolitis and 39 controls) (Table 1). Infants with bronchiolitis were significantly younger than the healthy ones (3.8 ± 3.0 months vs 6.1 ± 3.5, *P* = 0.002) and had significantly less frequent parental atopy (*P* = 0.017). From the 96 infants included in the study, 187 available samples were included in the analysis (94 fecal samples and 93 respiratory samples), although some samples did not successfully pass the sequencing step (mainly due to low microbial biomass and/or failure in the amplicon libraries and/or a low number of reads) (Fig. 1).

**TABLE 1:** Clinical data of the infants hospitalized due to bronchiolitis and the healthy control group^*a*^^,*b*^

Demographics	Bronchiolitis (*n* = 7)	Controls (*n* = 7)	OR (95% CI)	*P*
Age (months)	3.8 ± 3.0	6.1 ± 3.5		0.002
Male	31 (54.4%)	18 (46.2%)		
Prematurity	7 (12.3%)	3 (7.7%)		
Bronchiolitis episode				
Temperature > 37.9°C	23 (40.4%)			
Highest temperature	38.6 ± 0.6			
Hypoxia (sat O_2_ < 93%)	50 (87.7%)			
Chest X-ray infiltrate	9 (15.8%)			
Antibiotic therapy	8 (14%)			
Viral coinfection	20 (35%)	0		
Viral detection	51 (89.4%)	19 (48.7%)		<0.001
Outcome				
Duration of stay (days)	3.2 ± 0.4			
Duration of fever (days)	2.4 ± 1.7			
Duration of hypoxia (days)	3.9 ± 4.3			
PICU admission	2 (3.5%)			
Previous asthma risk factors				
Breastfeeding	70.7% (42)	66.7% (26)		0.492
Maternal asthma	17.5% (10)	10.3% (4)		0.398
Paternal asthma	14% (8)	7.7% (3)		0.517
Maternal atopy	10.5% (6)	40.0% (16)	2.3 (1.5–3.5)	0.001
Paternal atopy	10.5% (6)	28.2% (11)	1.8 (1.1–2.9)	0.029
Animals at home	21.1% (12)	15.4% (6)		0.595
Maternal smoker	12.3% (7)	12.8% (5)		0.963
Paternal smoker	24.6% (14)	17.9% (7)		0.615
Brothers < 5 years	43.9% (25)	35.9% (14)		0.525
Attends day care	35.1% (20)	20.5% (8)	1.4 (1.03–2.1)	0.043
Influenza vaccine	5.3% (3)	7.7% (3)		0.683
Atopic dermatitis	12.3% (7)	15.4% (6)	2.3 (1.5–3.4);	0.017
Allergic rhinitis	0	0		
Food allergies	3 (5.3%)	10.3% (4)		0.627
Long-term evolution				
Wheezing episodes	50.9% (29)	7.7% (3)	2.1 (1.5–2.8)	<0.001
No. of episodes	2.5 ± 0.4	0.5 ± 0.3		0.027
Respiratory admission	14% (8)	0	1.8 (1.5–2.1)	0.019
No. of admissions	0.4 ± 0.1	0		
Inhaled corticosteroids	6.5% (10)	2.6% (1)		0.234
Montelukast	5.3% (3)	0		0.266

^*a*^ OR, odds ratio; CI, confidence interval; and PICU, pediatric intensive care unit.

^*b*^ Quantitative data are expressed as means and standard deviation. OR is only added when it is significant.

### Gut and nasopharyngeal microbiota and factors shaping their composition and diversity

**Fig 2:**
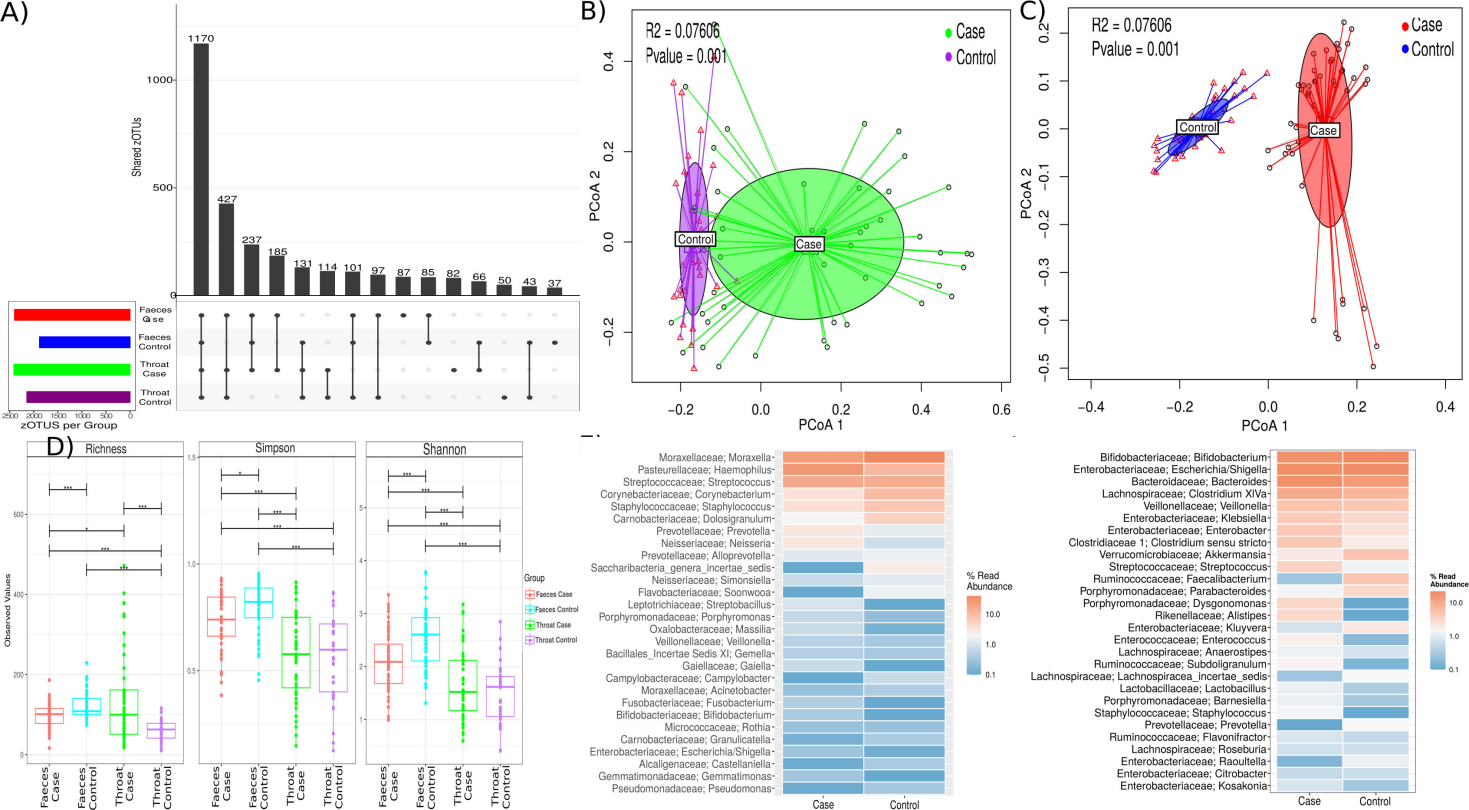
Taxonomy and alpha and beta diversity from nasopharyngeal aspirate and gut microbiota in the patients with bronchiolitis and in the controls. (**A**) Shows the amplicon sequence variants shared by all the study groups. (**B and C**) Principal coordinates analysis (PCoA) plot based on Bray–Curtis dissimilarities of nasopharyngeal (**B**) and gut (**C**) microbiota composition of samples from all patients included in the study. *P*-value corresponds to the Adonis2 PERMANOVA test. (**D**) Boxplot showing richness and Simpson and Shannon diversity indices according to the bronchiolitis and control groups in the NPA and gut microbiota of these patients. (**E and F**) Relative abundance of ASVs according to bronchiolitis in NPA (**E**) and gut (**F**).

A total of 13,334.437 million good-quality sequences were obtained from the study participants after bioinformatics processing (including length/quality filtering and denoising) and removal of contaminant sequences according to the criteria described in Materials and Method, representing a median of 77,659.92 sequences per sample (±17,656.72 SD). A mean of 2,208.5 ASVs by group (±62.89 SD) were extracted from these sequences, although the core microbiota was composed of 1,170 ASVs (Fig. 2A); however, the samples are highly variable, with 114 ASVs shared in the nasopharynx samples and 85 ASVs shared only by the stool samples. The shared samples in all patients with bronchiolitis were 185 ASVs, much higher than the 43 shared only by the controls, increasing the possibility of certain marker bacteria for the infants with bronchiolitis.

Using the Bray–Curtis dissimilarity matrix, significant differences were also found according to the respiratory health status, affecting both gut and NPA microbiota (Fig. 2B and C). In the NPA samples, the microbiota composition was found to be significantly different between the infants with bronchiolitis and the controls (*R*^2^ = 0.0716, *P* = 0.001). But we conducted the comparisons, not shown in figures, such as the comparison between the severity of bronchiolitis, specifically between severe and mild/moderate bronchiolitis (*R*^2^ = 0.094, *P* = 0.001), between those who developed recurrent wheezing during follow-up and those who did not (*R*^2^ = 0.0265, *P* = 0.035), and between those who had three or more recurrent wheezing episodes during follow-up and those who did not (*R*^2^ = 0.035, *P* = 0.040).

Interestingly, a dissimilarity in gut microbiota was also evident, being significantly associated with bronchiolitis (*R*^2^ = 0.026, *P* = 0.001), the severity of bronchiolitis (severe vs mild/moderate) (*R*^2^ = 0.731, *P* = 0.001), recurrent wheezing (*R*^2^ = 0.0183, *P* = 0.048), and three or more wheezing episodes during follow-up (*R*^2^ = 0.040, *P* = 0.020).

Alpha-diversity analyses showed statistically significant differences between gut and nasopharyngeal microbiota, as expected, and between the bronchiolitis and control groups (Fig. 2D). Bivariate analyses showed a significant decrease in gut microbial richness, Simpson, and Shannon diversity indices in the bronchiolitis group (infection group) compared with the controls (*P* = 0.001 in all indices). In the NPAs, an opposite trend was observed: infants with bronchiolitis had a higher and significative richness index (*P* = 0.001) than controls. For the Shannon and Simpson diversity indices, no statistically significant differences were observed between cases and controls.

### Bacterial biomarkers associated with respiratory health status

The most abundant genera in the NPAs of cases compared to controls were *Haemophilus* (27.8% vs 11.3%; *P* = 0.006), *Streptococcus* (27.8% vs 10.3%; *P* = 0.67)*, Prevotella* (3.2% vs 1.3%; *P* = 0.007), and *Neisseria* (3.2% vs 0.8%; *P* < 0.001). In contrast, a lower abundance of *Moraxella* (39.8% vs 23.6%; *P* = 0.04), *Corynebacterium* (5.5% vs 2.1%; *P* < 0.001), *Staphylococcus* (7.6% vs 3.18%; *P* = 0.540), and *Dolosigranulum* (17.4% vs 3.5%; *P* < 0.001) genus were found in cases than in controls (Fig. 2E).

In the gut, the distinct genera between the cases and the control group were *Escherichia/Shigella* (21.8% vs 18.8%; *P* = 0.33), *Bacteroides* (17.2% vs 14.2%; *P* = 0.31), *Clostridium* (10.4% vs 7.4%; *P* = 0.34), *Enterobacter* (4.2% vs 2.6%; *P* = 0.12), *Klebsiella* (3.7% vs 3.1%; *P* = 0.03), and *Streptococcus* (4% vs 2%; *P* = 0.92). In contrast, the least representative genera in cases compared to controls were *Bifidobacterium* (21.1% vs 20.3%; *P* = 0.35), *Faecalibacterium* (3.3% vs 0.3%; *P* < 0.0001), *Veillonella* (4.9% vs 4.8%; *P* ≤ 0.00102)*,* and *Akkermansia* (5.7% vs 3.7%; *P* = 0.03) (Fig. 2F).

**Fig 3:**
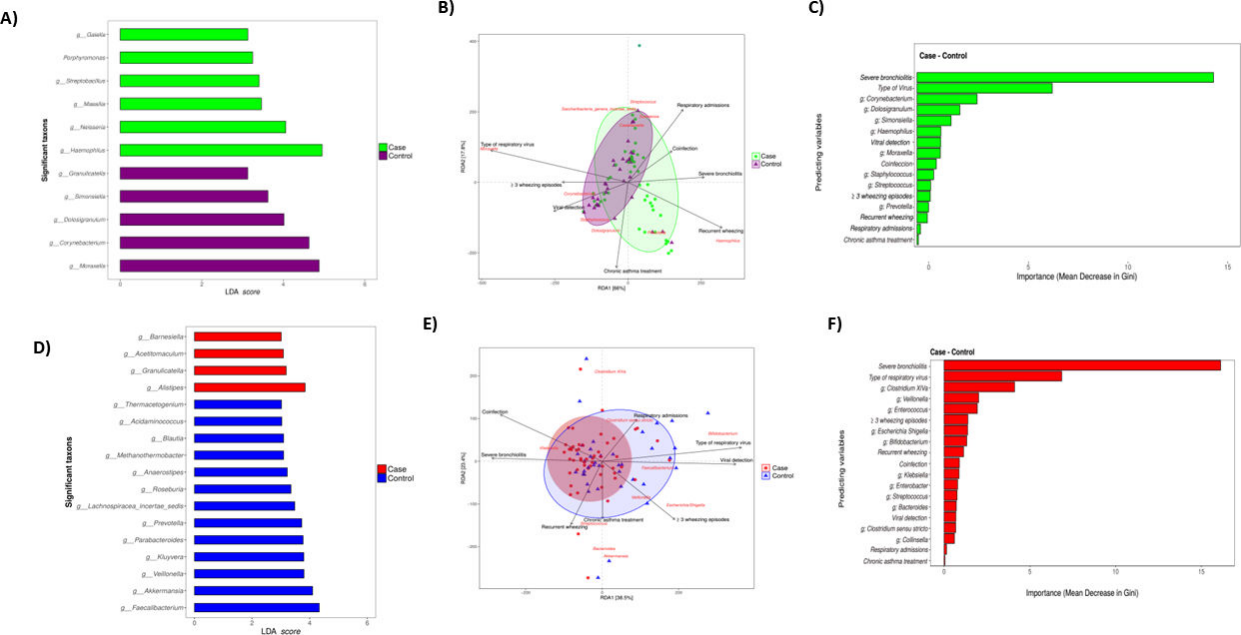
Statistically significant taxonomic change in bronchiolitis vs control samples in nasopharyngeal aspirate and gut. (**A and D**) Linear discriminant analysis (LDA) effect size in NPA (**A**) and gut (**D**). (**B and E**) Triplot of RDA showing the distribution of NPA (**B**) and gut (**E**) samples with reference to bacterial genera and explanatory variables. The ellipses are drawn containing 75% of each group of samples from each study group and colored accordingly. The arrows indicate the direction and strength (length) of the explanatory variables. The red names correspond to the higher abundance of each bacterial genus. (**C and F**) Performance of random forest (RF) classifier models. RF models were used for distinguishing bronchiolitis cases from controls in NPA samples (**C**) and bronchiolitis cases from controls in gut samples (**F**) through the representation of the mean decrease in the Gini index of each variable.

The statistical significance of the pathogens’ differential distribution according to respiratory health status was assessed with LEfSe. Among the most abundant taxons, LEfSe showed that the NPA microbiota of the infants with bronchiolitis (Fig. 3A) was characterized by the presence of *Haemophilus* (*P* = 0.006)*, Neisseria (P <* 0.001), *Massilia (P <* 0.001), *Streptobacillus (P <* 0.001), *Porphyromonas (P =* 0.040), and *Gaiella (P <* 0.001) The genera overrepresented in the control group were *Moraxella* (*P* < 0.001)*, Corynebacterium* (*P* < 0.001), *Dolosigranulum* (*P* < 0.001)*, Simonsiella* (*P* < 0.001), and *Granulicatella* (*P* = 0.024). In the gut samples (Fig. 3B), LEfSe identified *Alistipes* (*P* = 0.018), *Granulicatella* (*P* = 0.015)*, Acetitomaculum* (*P* < 0.001), and *Barnesiella* (*P* < 0.001) as overrepresented in the bronchiolitis group, whereas *Faecalibacterium* (*P* < 0.001), *Akkermansia* (*P* = 0.03), *Veillonella* (*P* < 0.001), *Kluyvera* (*P* = 0.01), *Parabacteroides* (*P* = 0.014), *Prevotella* (*P* = 0.007), *Lachnospiraceae incertae sedis* (*P* < 0.001), *Roseburia* (*P* < 0.001)*, Anaerostipes* (*P* < 0.001), *Methanothermobacter* (*P* < 0.001), *Blautia* (*P* < 0.001), *Acidaminococcus* (*P* < 0.001), and *Thermacetogenium* (*P* < 0.001) were characteristic of the controls’ microbiota.

### Nasal and gut microbiota profiles in infants with bronchiolitis and in healthy infants

Random forest models showed the predictions’ performance in distinguishing cases from controls by combining only microbiological parameters assessed in NPAs (Fig. 3C) and gut samples (Fig. 3F). Receiver operating characteristic curves of the models showed areas under the curve (AUC) of 0.9743 and 0.9773 for model I (NPA in case vs controls) and model II (gut in case vs controls), respectively. In model I, the presence of *Corynebacterium*, *Dolosigranulum*, *Simonsiella, Haemophilus*, *Moraxella*, *Staphylococcus*, *Streptococcus,* and *Prevotella* was predictively relevant (in the order of importance) in infants with bronchiolitis vs controls, as well as in infants with severe bronchiolitis, positive viral detection, coinfection, recurrent wheezing, frequent wheezing (≥3 wheezing episodes), respiratory admissions, and prescription for chronic asthma treatment (Fig. 3C). When performing gut analysis, *Clostridium XlVa*, *Veillonella*, *Enterococcus*, *Escherichia/Shigella*, *Bifidobacterium*, *Klebsiella*, *Streptococcus*, *Clostridium sensu stricto*, *Bacteroides*, *Enterobacter,* and *Collinsella* genera were more predictively relevant in infants with bronchiolitis than in the controls, as well as in infants with severe bronchiolitis, positive viral detection, viral coinfection, recurrent wheezing, ≥3 wheezing episodes, respiratory admissions, and prescription for chronic asthma treatment (Fig. 3F). These biomarkers (in the order of importance) make us differentiate or allow us to classify the most important factors between bronchiolitis and non-bronchiolitis.

### Microbiota profile and factors shaping composition and diversity

To observe the behavior of the significant variables that might influence beta diversity, they were projected onto a redundancy analysis with Bray–Curtis (Fig. 2B and E). In the NPA samples from the bronchiolitis group (Fig. 3B, upper left quadrant), *Dolosigranulum*, *Corynebacterium,* and *Staphylococcus* were associated with the type of virus identified (*P* = 0.006) and with the development of ≥3 wheezing episodes during follow-up (*P* = 0.02). As shown on the right side of the figure, *Haemophilus* was associated with recurrent wheezing and *Streptococcus* with severe bronchiolitis, viral coinfection, and respiratory admissions during follow-up (*P* = 0.03). *Neisseria* and *Saccharibacteria* genera *incertae sedis,* in the mean values of the graph, were associated with the prescription of chronic asthma treatment.

The gut microbiota findings from infants with bronchiolitis are shown in Fig. 3E. As shown in the lower right quadrant, the genera *Bifidobacterium*, *Faecalibacterium, Escherichia/Shigella, Veillonella,* and *Akkermansia* tended to be associated with the type of virus and recurrent wheezing (*P* = 0.091). In the lower left quadrant, *Streptococcus* and *Klebsiella* were associated with severe bronchiolitis, viral coinfection, and recurrent wheezing (*P* = 0.03). Moreover, respiratory admissions during follow-up were related to *Clostridium* species.

The results of the fuzzy set ordination and the cal_diff function are shown in Tables S1 and S2. In the NPA samples, the fuzzy set ordination found statistically significant results associated with severe bronchiolitis (*P* = 0.001), positive viral detection (*P* = 0.01), type of respiratory virus (*P* = 0.001), recurrent wheezing, ≥3 recurrent wheezing episodes (*P* = 0.05), and respiratory admissions (*P* = 0.05). In the gut samples, the fuzzy set ordination obtained the following significant factors: severe bronchiolitis (*P* = 0.001), type of respiratory virus (*P* = 0.05), recurrent wheezing, and ≥3 wheezing episodes (*P* = 0.05).

For the cal_diff in the NPA samples, significant results were associated with severe bronchiolitis (*P* = 0.001), viral detection (*P* = 0.001), type of respiratory virus (*P* = 0.001), coinfection (*P* = 0.05), recurrent wheezing (*P* = 0.001), ≥3 wheezing episodes (*P* = 0.001), respiratory admissions (*P* = 0.01), and chronic asthma treatment (*P* = 0.05). In the gut samples, the following significant factors were obtained: severe bronchiolitis (*P* = 0.001), viral detection (*P* = 0.001), type of respiratory virus (*P* = 0.001), recurrent wheezing (*P* = 0.001), ≥3 wheezing episodes (*P* = 0.001), and respiratory admissions (*P* = 0.05).

### Impact of viral coinfection during bronchiolitis

**Fig 4:**
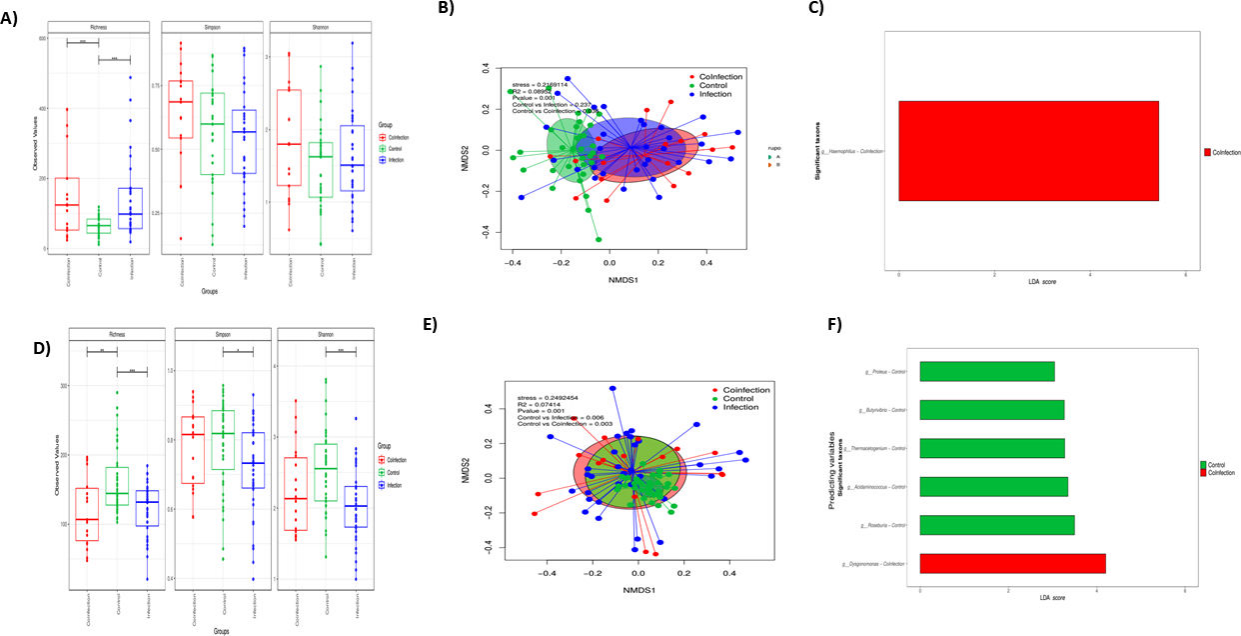
Effects of the co-infection variable on alpha and beta diversity and on taxonomy in the patients with bronchiolitis. (**A and D**) Boxplot showing richness, Simpson, and Shannon diversity indexes in NPA (**A**) and gut (**D**) microbiota with respect to the co-infection. (**B and E**) Principal coordinates analysis (PCoA) plot based on Bray–Curtis dissimilarities of nasopharyngeal (**A**) and gut (**B**) microbiota composition of samples from all study patients. *P*-value corresponds to the Adonis PERMANOVA test. (**C and F**) Linear discriminant analysis (LDA) effect size in NPA (**A**) and gut (**D**).

In NPA, the alpha-diversity differed significantly in the richness factor (*P* = 0.001) between the viral coinfection and the single infection group, on the one hand, and the control group on the other, but there were no statistically significant differences in the Simpson and Shannon indices (Fig. 4A). Regarding beta-diversity, significant differences were found between single infections and coinfections (PERMANOVA: *R*^2^ = 0.089, *P* = 0.001; Bray–Curtis distance and pairwise comparisons, *P* = 0.03) (Fig. 4B). LEfSe analysis reported an enrichment of *Haemophilus* genus in the infants’ NPA as a possible biomarker of viral coinfections (Fig. 4C).

In the gut, alpha diversity was also influenced by the detection of viral co-infection or single infection (*P* = 0.01 and *P* = 0.001, respectively) as shown in the richness and the Simpson (*P* = 0.05) and Shannon (*P* = 0.001) indices in the healthy group vs the infection group (Fig. 4D). Beta diversity also differed between single infections and coinfections (PERMANOVA: *R*^2^ = 0.07414, *P* = 0.001, Bray–Curtis distance and pairwise comparisons, *P* = 0.003 coinfection vs control and *P* = 0.006 infection vs control) (Fig. 4E). LEfSe analysis reported an enrichment of *Dysgonomonas* in the infants’ gut as a possible biomarker of viral coinfections (Fig. 4F).

### The impact of respiratory syncytial virus on the microbiota

**Fig 5:**
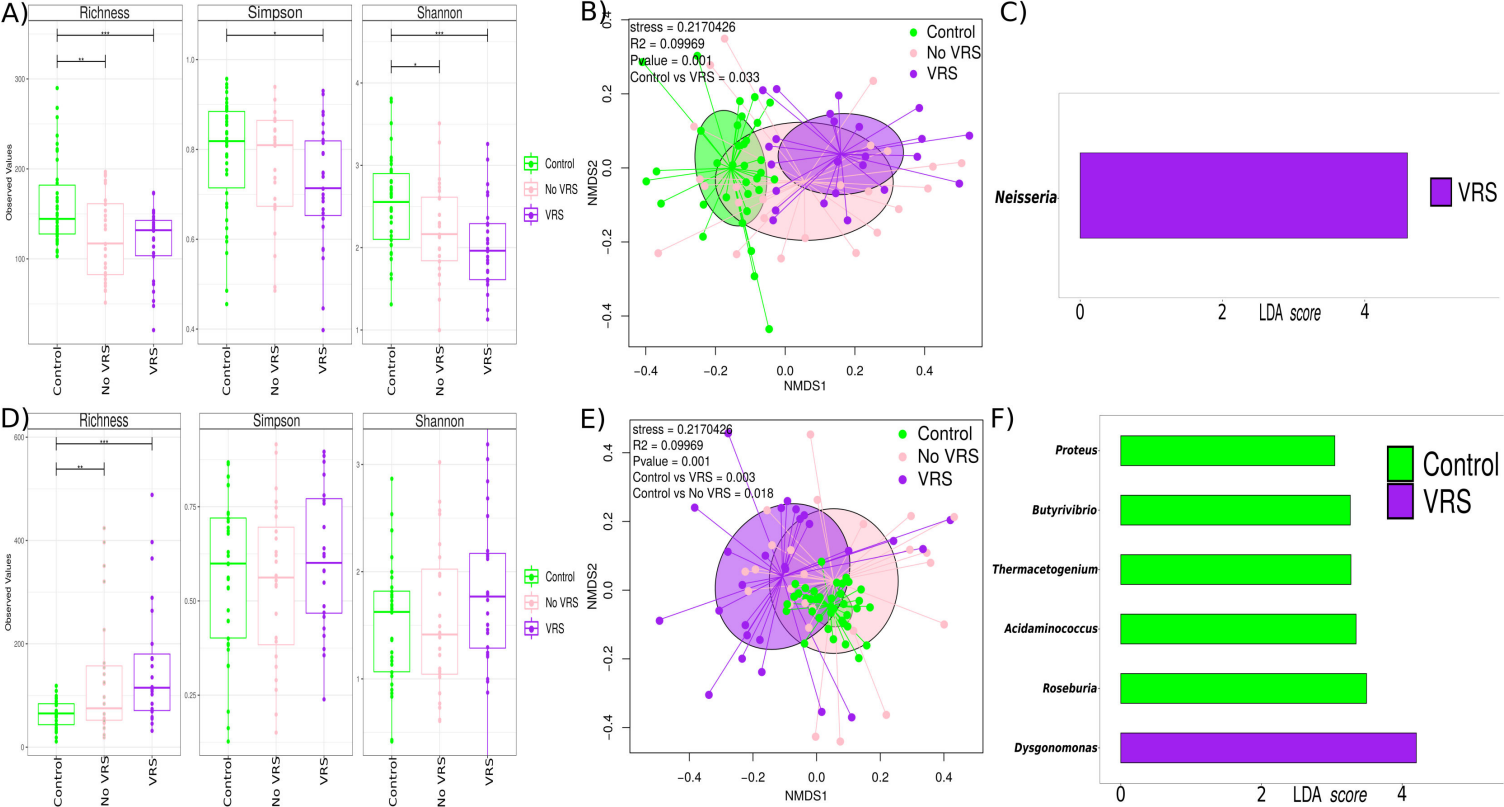
Effects of the respiratory syncytial virus infection on alpha and beta diversity and on taxonomy in the patients with bronchiolitis. (**A and D**) Alpha diversity boxplot (richness, Simpson, and Shannon diversity indexes) in NPA (**A**) and gut (**D**) microbiota. (**B and E**) Principal coordinates analysis (PCoA) plot based on Bray–Curtis dissimilarities in nasopharyngeal (**A**) and gut (**B**) microbiota composition of samples from all study patients. *P*-value corresponds to the Adonis PERMANOVA test. (**C and F**) Linear discriminant analysis (LDA) effect size in NPA (**A**) and gut (**D**).

RSV was the most prevalent virus associated with bronchiolitis in single infection or in coinfection with other viruses (Table S3). The impact of RSV on the microbiota was evaluated separately. In NPA, the alpha diversity differed significantly in richness when comparing the control group with the non-RSV bronchiolitis group (*P* = 0.01) and the control group vs the RSV bronchiolitis group (*P* = 0.001) (Fig. 5A). Beta diversity showed significant differences overall (PERMANOVA: *R*^2^ = 0.099, *P* = 0.001) but also in pairwise comparisons, specifically in controls vs RSV samples (*P* = 0.03) (Fig. 5B). LEfSe identified an enrichment of *Neisseria* genus associated with RSV in the NPA samples (Fig. 5C).

In the gut, the alpha diversity showed statistically significant differences in richness when comparing the control group vs the non-RSV group (*P* = 0.01) and the control group vs the RSV samples (*P* = 0.001), with higher diversity in the RSVs, followed by the non-RSV and control samples. In addition, similar differences were observed in the Shannon index, showing statistically significant differences only in the control group vs the RSV group in the Simpson index (*P* = 0.05) (Fig. 5D). Beta diversity showed statistically significant differences overall (PERMANOVA: *R*^2^ = 0.099, *P* = 0.001) and in pairwise comparisons, specifically in the controls vs the RSV samples (*P* = 0.003) and in the controls vs the non-RSVs (*P* = 0.018) (Fig. 5E). An enrichment of *Dysgonomonas* genus was identified by LEfSe as a possible gut biomarker linked to RSV (Fig. 5F).

## DISCUSSION

The role of the microbiota in the immune response of infants with respiratory infections is still unknown. The microbiota profile might be characteristically associated with viral infections, as shown in our study. The analysis of the gut and respiratory microbiota in hospitalized infants with bronchiolitis showed a different pattern from that of the healthy infants. Certain factors, such as the severity of the acute episode and the presence of RSV alone or in coinfection, were associated with specific patterns in both the gut and respiratory microbiota. In addition, children who were going to develop respiratory morbidity, with wheezing episodes in their first year after discharge, also had a characteristic microbiota profile at the acute respiratory infection, which could help to predict this progression and might support the idea that the intestinal and respiratory microbiota are a single organ (24).

In our study comparing 57 hospitalized infants with bronchiolitis with 39 healthy infants, we observed a decrease in the richness of the gut microbiota and conversely an increase in the richness of the respiratory microbiota in admitted infants compared with controls. Low gut microbiota abundance would be related to low immunoglobulin A production (25) and less expansion of regulatory T cells (Tregs) (26). In viral infections, Treg cells are involved in the process of viral clearance and in numerous aspects of the innate and adaptive immune response. The decrease in fecal diversity in infants has been associated with an increase in inflammation via the Th2 pathway and with dysbiosis, with a decrease in bifidobacteria that could even predispose children to asthma (27). *Clostridium XlVa, Veillonella, Enterococcus, Escherichia/Shigella, Bifidobacterium, Klebsiella, Streptococcus, Clostridium sensu stricto, Bacteroides, Enterobacter,* and *Collinsella* were increased in our infants hospitalized with bronchiolitis.

The increased diversity in the respiratory samples of our patients with bronchiolitis was clearly polarized toward microbiota profiles with an increase in certain bacterial families. Thus, these patients had an overrepresentation of *Gaiella, Porphyromonas, Streptobacillus, Massilia, Neisseria,* and *Haemophilus,* whereas in the controls, *Granulicatella, Simonsiella, Dolosigranulum, Corynebacterium,* and *Moraxella* were mostly observed, constituting a protective flora. Similar results to ours have been reported in the profile of nasal microbiota in a small number of children with bronchiolitis and in controls, although no differences in intestinal microbiota were detected (28). Also, Hasegawa et al. (11) identified similar profiles of respiratory microbiota in infants with bronchiolitis.

The viral agent identified in the bronchiolitis episode was one of the factors that most influenced our patients’ microbiota profile. The presence of RSV, single or in coinfection, was associated in our patients with intestinal dysbiosis and with an increase in *Dysgonomonas* identified by LEfSe as a possible gut biomarker linked to RSV. Simultaneously, increased alpha diversity richness was observed in the NPA samples, as well as a special increase in *Neisseria*, and in the case of coinfections, an important predominance of *Haemophilus.* The overrepresentation of *Haemophilus* in the respiratory microbiota of children with RSV bronchiolitis has been widely reported (28, 29) and could be involved in the severity and higher risk of recurrence of respiratory infections (30). Schippa et al. (31) found a higher representation of *Haemophilus* and *Streptococcus pneumoniae* in nasal samples from infants with RSV bronchiolitis, which was even greater in the most severe cases. In our series, the most severe cases were also associated with overrepresentation of *Haemophilus*. Our results, like those of Harding et al. (32), showed an alteration in alpha and beta diversity in patients with more severe bronchiolitis.

One of the most interesting aspects of our results is the possible association between the microbiota composition (intestinal and respiratory) of infants with bronchiolitis and the medium-term respiratory outcomes, mainly recurrent wheezing during the year of follow-up. A higher risk of developing asthma in children up to 5 years of age with a decrease in their intestinal microbiota diversity and richness has been suggested and could even be an early risk marker (33). Similar results were observed in our study in those infants who developed three or more wheezing episodes during the first year. As we previously mentioned, dysbiosis, and especially the increase in enterobacteria, leads to increased inflammation with Th2 pathway predominance that could play a role in the later development of asthma (34). Simultaneously, the respiratory microbiota could be protective when *Dolosigranulum* and *Corynebacterium* are the predominant profile; in contrast, a high relative abundance of *Moraxella*, *Haemophilus,* and *Streptococcus* has been associated with an increased risk of recurrent respiratory infection, inflammatory sequelae, and even asthma (30). In our patients, a relationship was observed between the presence of these bacteria (*Haemophilus, Streptococcus, Neisseria,* and *Saccharibacteria*) and a poorer mid-term respiratory progression, with the development of recurrent wheezing in the months following bronchiolitis, especially with the presence of ≥3 wheezing episodes, suggesting a more severe outcome. This pattern has also been associated with asthma in other studies. Hilty et al. (35) conducted a systematic study on microbiota in the nasopharynx, oropharynx, and lower airways of children with asthma. The asthmatic group was characterized by a relative abundance of *Haemophilus* and *Staphylococcus* spp. (Proteobacteria) and reduced *Prevotella* spp. (Bacteroidetes) compared with controls (35). Our patients were young and could therefore not yet be accurately diagnosed with asthma. It is worth highlighting, however, that according to the International Consensus on Pediatric Asthma (36), the diagnosis of asthma for young children in whom functional testing is difficult to perform could be reached by the presence of ≥3 episodes of persistent wheezing and/or coughing, in a situation in which asthma is probable and other less common diseases have been ruled out. It would be interesting to re-evaluate our patients in 4–5 years and check whether the microbiota pattern has been maintained and whether the diagnosis of asthma can be confirmed.

It is important to highlight the impact that our results could have if they were confirmed. If gut and respiratory dysbiosis could increase the risk of developing asthma in infants admitted for bronchiolitis, a therapeutic strategy based on the oral administration of prebiotics or probiotics, aimed at correcting the microbiota impairment and the related dysregulation, could be explored. Even an intranasal approach of applying bacterial components (such as endotoxin and flagellin) could be of interest, as shown by a number of animal models (37).

Our study has several limitations. First, the number of patients included was relatively small; however, they were very homogeneous and clinically well characterized. We cannot draw conclusions about the role of respiratory viruses other than RSV, given that they are under-represented since our work fundamentally covers two winter epidemics. We consider that the observed changes in the microbiota were possibly caused by the respiratory viral infection; however, we cannot rule out that they occurred before the infection or that other factors, such as the older age of the control group, could have had an effect. Despite the age difference, most of the children were younger than 6 months and had not been introduced to complementary feeding. In addition, it is important to consider the significantly higher risk of bronchiolitis in those attending day care (where different levels of microbiota transmission/exchange could occur). However, confounding factors such as antibiotic therapy were ruled out and, given the young age of our study’s participants, the diet was very similar in all of them, with a high proportion of breastfeeding. The simultaneous study of the intestinal and respiratory microbiota makes our study interesting, as does the follow-up of these children.

In summary, our study reported a clearly distinct respiratory and intestinal microbiota pattern in infants with bronchiolitis compared with controls. A lower richness in the intestinal microbiota and an increase in the diversity of the respiratory microbiota (but with an increase in bacteria such as *Haemophilus, Streptococcus,* and *Neisseria*) were observed in infants with bronchiolitis, in those with the most severe symptoms, and in those who subsequently developed recurrent wheezing episodes in the year after discharge. The presence of RSV, alone and in coinfection, was the main contributor to the microbiota differences. Longer-term follow-up of these children could help clarify the role of this microbiota pattern as a possible predictor of asthma after admission for bronchiolitis.

## Data Availability

The data are publicly available from the original study: European Nucleotide Archive accession number PRJEB71847.

## SUPPLEMENTAL MATERIAL

Table S1 spectrum.02556-23-s0001.docx FSO with the significant variables in NPA and gut samples.

Table S2 spectrum.02556-23-s0002.docx Test of the difference in environmental variables across groups with the cal_diff function.

Table S3 spectrum.02556-23-s0003.docx Viral detection.

ASM does not own the copyrights to Supplemental Material that may be linked to, or accessed through, an article. The authors have granted ASM a non-exclusive, world-wide license to publish the Supplemental Material files. Please contact the corresponding author directly for reuse.
