# Supplementary material for: Gut and respiratory tract microbiota in children younger than 12 months hospitalized for bronchiolitis compared with healthy children: can we predict the severity and medium-term respiratory outcome?
Source: Microbiol Spectr. 2024 May 24;12(7):e02556-23. doi: 10.1128/spectrum.02556-23 (PMC11218511; doi:10.1128/spectrum.02556-23)

Table 1 (supplementary file). Fuzzy Set Ordination (FSO) table with the significant variables in NPA and gut samples.


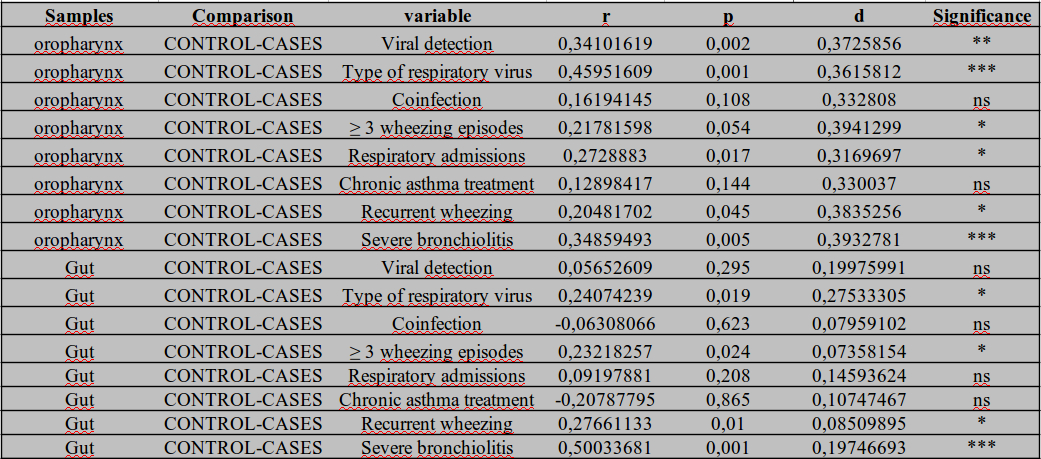

Supplement: Table S1 — FSO with the significant variables in NPA and gut samples. [file spectrum.02556-23-s0001.docx]
