# Supplementary material for: Gut and respiratory tract microbiota in children younger than 12 months hospitalized for bronchiolitis compared with healthy children: can we predict the severity and medium-term respiratory outcome?
Source: Microbiol Spectr. 2024 May 24;12(7):e02556-23. doi: 10.1128/spectrum.02556-23 (PMC11218511; doi:10.1128/spectrum.02556-23)

Table 2 (supplementary file). Test of the difference in environmental variables across groups with the cal_diff function.


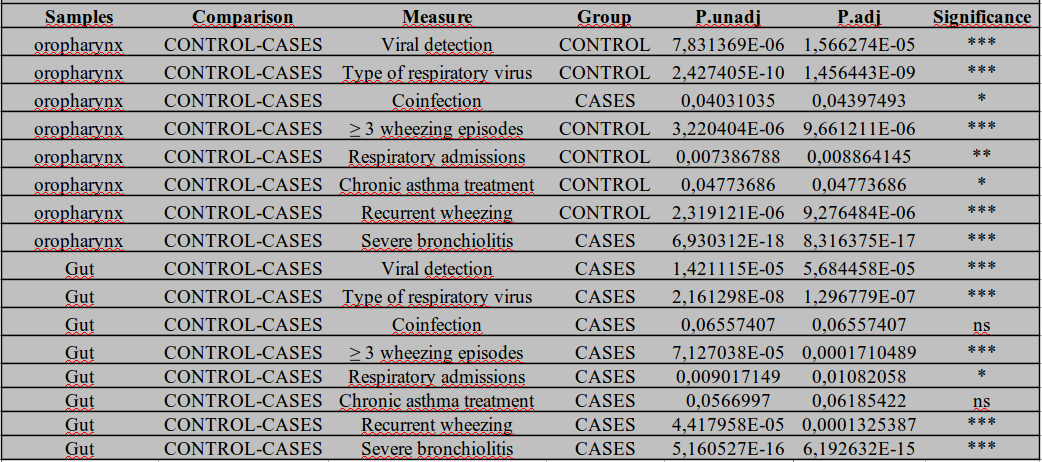

Supplement: Table S2 — Test of the difference in environmental variables across groups with the cal_diff function. [file spectrum.02556-23-s0002.docx]
