# Supplementary material for: Gut and respiratory tract microbiota in children younger than 12 months hospitalized for bronchiolitis compared with healthy children: can we predict the severity and medium-term respiratory outcome?
Source: Microbiol Spectr. 2024 May 24;12(7):e02556-23. doi: 10.1128/spectrum.02556-23 (PMC11218511; doi:10.1128/spectrum.02556-23)
Supplement: Table S3 — Viral detection. [file spectrum.02556-23-s0003.docx]

Table 3 (supplementary file). Viral detection in bronchiolitis cases and controls

**Virus Bronchiolitis (N=57) Controls (N= 39)**

Negative 6 20

RSV 19 0

Rhinovirus 9 13

Influenza 1 2

HCoV 0 3

HAdV 0 1

PIV 1 0

hMPV 1 0

Coinfections 20 0

RSV+ HRV 7

RSV+ HCoV 2

HRV + hMPV 4

Other combinations 7

RSV: respiratory syncytial virus; HCoV: human coronavirus; HadV: human adenovirus; PIV: parainfluenza virus; hMPV: human metapneumovirus; HRV: human rhinovirus
